# Towards improved understandings of medical manslaughter: fatal medical error, accountability, and wider paths to learning

**DOI:** 10.1093/medlaw/fwag031

**Published:** 2026-08-13

**Authors:** Danielle Griffiths

**Affiliations:** School of Law and Social Justice, University of Liverpool, Liverpool, L69 7ZS, United Kingdom

## Abstract

Criminal liability for fatal errors made by healthcare professionals, medical manslaughter (MM), has prompted much academic and professional debate. There is concern that doctors are increasingly vulnerable to this offence when they make inadvertent clinical errors in complex healthcare systems where mistakes are common and often arise from systemic causes. Yet, these discussions have been dominated by a few appellate court cases and focused on doctors, not victims. This highlights a key data gap and suggests that there may be patterns in MM cases not fully understood. This article analyses 192 MM cases that constitute all known investigations referred to the Crown Prosecution Service between 2007 and 2018. The analysis shows that MM prosecutions are extremely rare but also reveals previously unexplored trends, including high levels of individual advertent faults and deeper structural concerns beyond systemic faults. Although MM remains a contentious offence, attention must also shift to this wider body of cases, which demand approaches extending beyond criminal law and capable of addressing cross-cutting forms of culpability including structural violence. By situating these cases within a broader regulatory context, I demonstrate that MM cases constitute a vital yet underutilized resource for learning and accountability.

## I. Introduction

In the UK, the National Health Service estimates that there are approximately 11,000 avoidable deaths annually owing to patient safety incidents (PSIs).^1^ A proportion of these deaths include medical errors committed by healthcare professionals (HCP).^2^ Death as a result of medical error signifies the point at which a wider array of investigatory processes become possible and legally necessary, particularly in relation to referral to a coroner.^3^ The most serious fatal errors are also referred to the police for a possible charge of gross negligence manslaughter (GNM) referred to as ‘medical manslaughter’ (MM) when applied to HCPs. The offence of MM is committed where a death is the result of a grossly negligent (though otherwise lawful) act or omission on the part of the defendant.^4^ Yet, the use of MM to legally address fatal medical error has led to concerns about the fairness and effectiveness of prosecutions.^5^

MM is a notoriously vague offence.^6^ While it is now recognized that rates of MM prosecutions are low,^7^ there is concern that doctors are still vulnerable to the vagueness of MM when they make inadvertent clinical errors in complex and underfunded healthcare systems where errors are frequent and often have systemic as well as human causes.^8^ A culture of ‘toxic fear’ in relation to criminal charges has developed amongst medical professionals^9^ and led to law reform proposals that would offer better protection to HCPs and shift liability from individual inadvertent clinical errors to systemic culpability.^10^ Despite some recent clarity in the law around GNM, concern remains pervasive and legal scholars continue to attest to the unfairness of the rules governing the offence, their application and the need for reform to more effectively and fairly address fatal medical error.^11^

Yet, there is a data gap within this work. Due to difficulties in accessing data on MM cases, prevailing discourse around MM is largely informed by doctrinal analysis of a small number of contentious cases involving clinical errors made under pressure that have reached the appellate courts.^12^ Relatedly, work has also largely focused on the impact of the offence on HCPs, not victims.^13^ Where empirical work has been conducted, the research has used small unrepresentative samples, and also largely not centred the victims involved.^14^ This article offers a novel and evidence-based framework for a more comprehensive understanding of MM cases which can enhance effective learning and accountability. By extending analysis beyond contentious appellate court cases and small samples, I aim to bridge this data gap by analysing the nature of error and culpability across a broader set of MM cases. I use never previously accessed details of 192 MM cases which constitute all known investigations referred to the Crown Prosecution Service (CPS) between 2007 and early 2018.^15^ They illustrate that MM investigations provide essential untapped data on some of the most serious errors and highlight the need for broader forms of redress and reform.

The article is set out as follows. Section II outlines the methods used to gather the dataset of MM cases. Section III outlines the development of GNM and its use in a healthcare context. It traces the fears generated by the offence and assesses evidence for some of the key claims made. Section IV presents the data analysis. This confirms that the vast majority of cases do not proceed to court and shows that rates have declined further in recent years. It also shows that while some MM cases indeed arise from inadvertent clinical errors made under systemic pressures, many others reveal deeper previously unrecognized issues. Many cases show high levels of advertent fault in non-hospital settings, such as prisons, mental health and care home settings, and involve the neglect and abuse of vulnerable groups. These cases reveal forms of culpability that need to be addressed irrespective of whether they proceed to prosecution, particularly given that the vast majority no longer proceed to trial. They also highlight the need for a greater focus on victims and on the structural violence that contributes to such deaths. Section V situates the cases in the broader context of healthcare regulation and death oversight, including inquests and public inquiries, showing that such processes are often fragmented and lack proper means for learning and enforcement. It shows that more consistent and effective accountability, learning and justice in fatal medical error deaths could be achieved if data on all investigations of MM were collated as part of a national dataset for learning alongside the proceedings from other post-death inquiry and regulatory processes.

I make two main arguments. First, that scholarship on MM should examine a broader range of cases and move beyond its predominantly doctrinal focus. Analysis of the cases demonstrates that concerns extend beyond the application of the offence itself to the absence of effective mechanisms for learning, both where cases do not proceed and where they do. Secondly, approaches are needed that can address and prevent the cross-cutting structural dimensions of culpability, alongside individual and systemic failures within such a wider body of cases. I show that MM case files are a rich but unexplored source of data on fatal medical error that could form part of such a wider approach to learning. In making these arguments, I concur with previous work on the need to shift away from individual blame in the majority of MM cases, to address their wider causes. However, by grounding this argument in the evidence base provided by all MM cases, demonstrating how wider causes extend well beyond systemic failures, and linking these factors to broader bodies of research on healthcare regulation and post-death inquiry, I make a distinct contribution to the debate. I offer a more empirically grounded basis for understanding how such cases can be addressed in a manner that ensures fairness, accountability, and learning for victims and HCPs alike.

## II. Methods and scope of the research

Past work demonstrated that MM cases are referred to the police by a coroner, family, and/or hospital when there is reason to suspect criminal negligence in the care provided to the deceased.^16^ Thus, they represent a dataset of the most serious instances of fatal medical error, considered so severe that they were referred to the police. As will be shown in Section V, knowledge of some of these fatal error cases would not have emerged without the initiation of a criminal investigation, as while they should also be referred to a coroner, this does not always happen.^17^ Due to the complexity of MM, once a case has been opened, the police are advised to involve the CPS’s Special Crime and Counter Terror Division (SCCTD) at an early stage.^18^ Specialist prosecutors work closely with the police in a dynamic process that often involves medical witnesses and specialist barristers commissioned to provide independent advice. Thus, due to the unique nature and resourcing of these investigations, MM investigations often contain richer detail compared to other sources on medical error, such as PSI data, which are often limited and fragmented, as will be explored in the last part of the article. Consequently, MM cases form one of the most complete and discrete datasets available on serious fatal medical errors.

During Phase 1 of this research in 2018, the author co-led fieldwork with Oliver Quick. As part of this fieldwork, the SCCTD provided a table of 192 MM cases (190 and 2 prosecuted cases added later), comprising all known MM investigations handled by the CPS between 2007 and early 2018.^19^ This table of cases contained very basic information about each case: suspect name, date of incident, and the outcome, which allowed us to measure the number of referrals of MM cases over time, as well as the number of prosecutions, both of which have been notoriously difficult to ascertain. Analysis of these basic data can be found in Griffiths and Quick,^20^ which breaks down the table of cases to show numbers of prosecutions and investigations over time, the profession of the healthcare staff prosecuted, and the time taken for some of the cases to be disposed of.

This article builds significantly on that past work by drawing on data never previously systematically collated or analysed. Only 50 cases of the 192 cases contained a more detailed description of the error in question, where it occurred, and the type of healthcare professional investigated; such data was incomplete in the remaining 142 cases. In phase 2 of the research, I collated this missing information on suspect type, setting of error and nature of error/culpability for the remaining 142 cases in the table (so 192 cases in total) by using publicly accessible data. Many of these cases were reported online (in national and local newspapers) and had Prevention of Future Death (PFD) reports, inquest hearings and public inquiries attached to them, thus allowing for an online analysis to supplement the data in the table. Thus, the CPS’s case table supplied the initial data needed to assemble a more detailed and complete collection of publicly available information. The collation of further data would have been impossible without the individual case names in the CPS’ table, as a general search for MM cases in the public domain would not have produced decipherable or reliable results with a clearly defined scope. It is acknowledged that online news reports will contain selective coverage and more inaccuracies than data gathered directly from the CPS itself,^21^ yet more robust news sources were used (ie, not blogs or social media) and sources were cross-checked with other available online news sources as well as previously held data on inquests in MM cases,^22^ and previously held data on MM cases^23^ to ensure greater accuracy. In addition, information gathered from these online sources included more factual and uncontroversial details (type of HCP, place of death and brief details of what the error was) that are likely to be more accurately reported on online sites. Case details beyond the type of HCP could not be found in 47 of the 192 cases (24 per cent). Given these limitations, the article makes no claim that the dataset is wholly representative or capable of providing definitive conclusions about all MM cases. Rather, it reflects the methodological constraints of researching an area where access to official data is extremely limited. Nonetheless, the article offers a more nuanced understanding of MM cases, highlights the need for more systematic and transparent data collection in this field, and opens important avenues for further empirical and interdisciplinary research.

## III. Medical manslaughter: the development and use of a contested offence

### A. Gross negligence manslaughter

The current test for GNM derives from the ruling in *Adamako*.^24^ Lord Mackay set out four elements that need to be proven.

i) The existence of a duty of care to the deceased;
ii) Breach of the duty of care;
iii) The breach caused (or significantly contributed to) the death of the person; and
iv) The breach that caused the death was ‘grossly negligent’.^25^

Commentators have been critical of the judgment.^26^ The test is objective; subjective disregard and recklessness are not required for conviction.^27^ In addition, the fourth element of the test is vague and circular; prosecutors, experts and judges struggle to define ‘gross’ and juries are effectively directed to convict if they believe a crime has been committed.^28^ However, the GNM test has recently been the subject of significant judicial developments that have clarified the test and raised the bar of liability.^29^

First, the requirement that the breach of duty must pose a risk of death was considered and narrowed by the Court of Appeal in *Rose.*^30^ Honey Rose, an optometrist, failed to examine the back of a patient’s eyes during a routine eye test, which meant she missed symptoms of acute hydrocephalus; the patient’s condition went undiagnosed, and he died five months later. Rose was convicted of MM and appealed. The Court of Appeal, in a judgment delivered by Sir Brian Leveson, quashed Rose’s conviction and modified the test in *Adomako*, so that liability depends upon a breach of an existing duty of care giving rise to a reasonably foreseeable serious and obvious risk of death.^31^ It was held that a remote risk of death may have been reasonably foreseeable due to failure to carry out the test in an otherwise healthy patient, but that was not the same as a ‘serious and obvious risk’. The court set out that the test is both objective and prospective; hindsight is irrelevant.^32^ Following *Rose, Broughton* set out two additional elements to the four-stage test in *Adamako*; first, at the time of breach there must be a serious and obvious risk of death, and secondly it must be reasonably foreseeable at that time that the breach gives rise to a serious and obvious risk of death.^33^ While still an objective test, Stark argues that the test for GNM is now closer to more subjective fault elements found in other offences,^34^ focusing on the defendant’s beliefs and what conclusions about risks they could reasonably be expected to draw from them.^35^

The second tightening of the law concerns the description of the type of conduct that may amount to GNM. In *Sellu*, the Court of Appeal stated that to distinguish gross negligence, it is necessary to separate serious or very serious mistakes from conduct that is truly exceptionally bad.^36^ This emphasized the extremely high bar for liability. Fears over how this vague test is applied in prosecutorial decision-making prompted CPS guidance on MM and the factors likely to have a bearing on culpability in these cases.^37^ These include: ‘where there is a course of conduct by an individual and a series of serious breaches’; ‘the deliberate overriding or ignoring of systems which are designed to be safe and have proven to be safe’; or ‘when an individual acts against the advice of other members of the team alerting them to serious dangers or risk’.^38^

Despite greater clarity in the law, wider criticisms of the offence remain. Regardless of the subjectivity *Rose* injected,^39^ the mens rea for GNM is still largely determined by an objective test. Inadvertent error can still lead to conviction, and the offence lacks the subjective moral blameworthiness normally required for homicide.^40^ Moreover, after *Rose* some cases involving egregious negligence may now be less likely to result in criminal liability where the defendant was unaware of the relevant risk due to their negligence, compared to cases in which the defendant has properly engaged with their duty and thereby become aware of that risk but then failed to act.^41^ As Mullock argues, it is ‘incongruous to assess a competent professional who is simultaneously in a position of negligent ignorance’.^42^ While the thresholds have been raised and the tests somewhat clarified, GNM remains problematic.^43^

### B. Medical manslaughter

Academic and professional attention to GNM has grown alongside the use of the offence in a healthcare context, that is, the application of MM. Sustained debate and campaigning on MM was prompted in 2006 by Ferner and McDowell’s conclusion that there had been a dramatic increase in prosecutions against doctors.^44^ Although the Williams Review and data from Griffiths and Quick show that cases are actually very low, equating to less than one prosecution per year,^45^ fear persists. As inadvertent errors can nonetheless give rise to criminal liability, there is concern that professionals have limited control over their exposure to the criminal process. In response to these concerns, there have been arguments to replace the test of gross negligence with one of subjective recklessness so that only errors where there is clear advertence and subjective awareness of risk are prosecuted.^46^ However, research has shown that, particularly in recent decades, MM cases are rarely prosecuted without some evidence of subjective recklessness.^47^ Moreover, as will be discussed in Section IV, the objective/subjective dichotomy is not always clear,^48^ and subjective recklessness can still be found even when a HCP denies consciously taking a risk, so defendants in some of the controversial cases such as *Rose* could still be convicted under a subjective test.^49^ Nevertheless, these arguments have been persuasive in terms of offering some more protection to HCPs when they make inadvertent errors and offering more clarity in the legal test.

The above concerns are heightened given how inadvertence can arise under systemic pressure, further limiting individual control. Such fears peaked with the case of *Bawa-Garba* in 2016.^50^ Bawa-Garba was convicted of MM for misdiagnosing and failing to properly reassess 6-year-old Jack Adcock, who subsequently died of a cardiac arrest due to sepsis. Her application for leave to appeal against the conviction was rejected, and the General Medical Council called for her removal from the medical register, prompting mass furore and a flurry of academic work.^51^ She successfully overturned this decision to remove her from the medical register^52^ amidst much discussion over the systemic pressures Bawa Garba experienced on the day she made her fatal errors, including staff shortages and technical failures.^53^ It is often assumed in the academic literature that most other MM cases involve similar systemic issues and thus that errors are not the fault of the individual alone.^54^ Scholars question the extent to which such issues are taken into account in all stages of the criminal process, from prosecutorial decision-making to the trial process.^55^ Previous research has shown that systemic issues are in fact given due weight in prosecutorial decision-making, yet cases can still proceed to prosecution. In other words, the presence of systemic factors does not always mean high levels of individual fault are completely negated.^56^ Yet, how these factors are weighed up within a trial is less clear.^57^ In order to address such potential unfairness, arguments focus on the need to move away from taking an individualized adversarial approach to instead address the systemic faults that are deemed to underlie the clinical errors thought to dominate these cases, for example, using patient safety mechanisms.^58^

Such continued uncertainty surrounding the use of MM has had ramifications extending beyond professional and scholarly debates.^59^ Responding to rising levels of concern and campaigning, the Williams Review^60^ and Hamilton Review^61^ were commissioned by the Secretary of State for Health and General Medical Council, respectively, to look at the use of MM. Fear is also argued to be related to the wider shifts in GNM itself; some state that the significant decision in *Rose* was related to concerns by the Court of Appeal over the adverse impact of the offence on doctors.^62^ This led to concerns about what has been termed a ‘medicalisation of the law’,^63^ which Lilleker notes casts doubt on the true breadth of the offence.^64^ Relatedly, Laird argues that it is highly problematic that an offence that can be committed by anyone now appears to be being developed solely by reference to its impact upon HCPs.^65^ Such a skewed development in the offence is of particular concern when it is recognized that while *Rose* clarified the law to some extent, it has also led to negative consequences.^66^

Yet, there is a significant data gap in much of the above debate. Most work is based on a doctrinal analysis of contentious appellate court cases. Such a data gap suggests that fears may extend beyond the reality of these reported cases, that underlying patterns are not yet fully understood, and that further reforms to ensure learning and accountability beyond those currently proposed may be necessary.^67^ Indeed, the Hamilton Review concluded that ‘some of the anecdotes and allegations that we have heard during the course of this review have not been borne out by the facts’.^68^ The Review also noted that the whole review arose from a single case, Bawa Garba. There is thus an urgent need to bridge this data gap and interrogate where culpability lies in most cases. Additionally, within this work, the prosecution and investigation of doctors dominates discussions within academic papers, as well as policy reviews, with nurses rarely mentioned.^69^ As shown above, much of the debate is limited to a few Court of Appeal cases involving a doctor. It is unclear how far other HCPs are being drawn into the criminal process. Finally, there is insufficient exploration of victimhood, including whether certain groups are disproportionately affected by fatal medical errors.

To get a stronger understanding of trends in MM, it is necessary to examine all cases of MM referred to the CPS in order to analyse all first-instance cases and those which are not prosecuted. While these latter cases do not proceed as far as the high-profile appellate cases such as *Bawa-Garba* and *Rose* explored above, this does not mean they cannot provide valuable information about the prevalence and types of serious errors resulting in consideration of MM charges, as well as the severity and forms of culpability. Research on prosecutorial decision-making in MM cases has shown that difficulties in establishing causation, the high threshold needed for ‘gross’ negligence, and consideration of mitigating systemic factors may mean that even with evidence of ‘truly, exceptionally bad’ conduct, a case will not necessarily proceed to court.^70^ Yet, they can still indicate serious error and harm that needs to be learnt from. Hence, a key argument of this article is that reform proposals need to be based on data drawn from all cases of deaths arising from serious fatal medical error.

## IV. An empirical understanding of medical manslaughter

### A. Rates of investigations and prosecutions

Only 12 (6 per cent) of the 192 cases referred to the SCCTD were prosecuted. The Griffiths and Quick report based on our first phase of data showed that apart from one anomalous spike in 2014, prosecution rates have been very low and steady, equating to less than one per year.^71^ Since January 2018, these rates have declined further, and there have been just three MM prosecutions,^72^ which arguably reflects the raised and clearer thresholds post *Rose* and *Sellu.*^73^

In terms of rates of investigations, there was a rise in the rate of referrals to the SCD per year, rising slowly from 1 case in 2007 to 44 cases in 2014.^74^ While this rise declined to seven cases in 2018, the data do show an increase. Coroners, police and families are now much more likely to refer medical cases for criminal investigation due to wider factors such as increased awareness of medical errors and well-documented cases of mistreatment and negligence within NHS settings.^75^

Overall, these data indicate that prosecutions for MM have remained extremely low, declining further over recent years. This underlines that prosecutions are not the major concern, particularly after changes in the test for GNM. In contrast, criminal investigations into MM have increased over the past 20 years. Thus, despite growing demand for accountability in fatal medical error cases, the data show that the offence is ill-suited to addressing the types of culpability and typical errors typically made, as the vast majority of cases do not meet the evidential threshold for prosecution. However, without information about the nature of the errors across all cases, it is impossible to determine what the most appropriate form of accountability would be to ensure both learning and justice are achieved. Thus, in the final section of this paper, the types of error and culpability will be aggregated across all cases in order to analyse what wider lessons can be learnt from MM cases beyond a potential or actual criminal conviction.

### B. Who are the suspects and the deceased in medical manslaughter cases?

The dataset comprises 192 cases. Because some of the 192 cases included multiple suspects, there were 290 suspects overall. Suspects included 54 organizations, which were investigated under potential corporate liability: these comprised 40 hospitals, 11 care homes, a prison, and 2 regional ambulance services. Thus, the data demonstrate that corporate entities are frequently implicated in these investigations. However, the significant evidential difficulties involved in bringing charges against hospital trusts have been well documented.^76^ Of the 236 individuals investigated, there were 109 doctors (of whom 16 were GPs), 107 nurses, 9 cases in which it was unclear who the suspect was, or the database stated there was no suspect identified (since MM investigations often involve multiple suspects from the start), 1 dentist, 1 ophthalmologist, 5 pharmacists and 4 paramedics.

In terms of case outcomes, in the prosecuted cases there were 18 individual HCPs involved (12 cases overall); 10 doctors, 7 nurses, and 1 ophthalmologist.^77^ Thus, while there have been more doctors than nurses ending up in court and being investigated, there is not a substantial difference.

To date, analysis of disparity involving suspects in MM cases has been limited to considering the race of doctors most likely to be charged with MM.^78^ Recent work by Alghrani and Saad discusses how racial bias can seep into MM prosecutions due to the vagueness of the offence and discretion it allows.^79^ However, they, and others including the Hamilton Review,^80^ acknowledge that there is little hard data to support this. In MM cases, biases are one of many factors that influence whether a case proceeds, including age and vulnerability of the victim, whether a coroner or family refer a case, etc, and it is almost impossible to disaggregate and prove a single causal factor in such a small number of prosecutions.^81^ The CPS data did not contain enough detail on the race of suspects/defendants to draw any robust conclusions around disparity in investigations. Race was identifiable in the prosecuted cases, where 10 of the 18 (56 per cent) individuals prosecuted were BAME. However, this needs to be interpreted in the context of BAME groups making up nearly 50 per cent of hospital and community health services doctors and 41 per cent of all hospital and community health services doctors, nurses, and health visitors.^82^

There is some more robust evidence of disparity in case outcomes between doctors and nurses. As shown above, nurses are almost as likely to be investigated and prosecuted for the offence as doctors. Yet, all reported cases that have reached the Court of Appeal have involved doctors, not nurses, suggesting that nurses are less likely to challenge a conviction or a decision to strike off. There could be multiple explanations which warrant further investigation, but one reason could be that medical organizations such as the British Medical Association have greater resources and are more likely to be able to campaign against the criminalization of their members. The David Sellu appeal generated over £50,000 from fellow medics to crowd-fund a successful appeal against conviction,^83^ while doctors raised over £200,000 for legal assistance in Bawa Garba’s appeal against GMC erasure.^84^ Significantly, nurse Isabel Amaro, who was convicted alongside Bawa Garba, did not appeal her criminal conviction despite similar mitigating factors,^85^ and failed in her bid to challenge professional erasure. Amaro was not legally represented in her tribunal, whereas Bawa-Garba had a very experienced legal team due to the crowdfunding.^86^

A further disparity between nurses and doctors is that over 60 per cent of the cases involving nurses are neglect-type errors (see next section) in settings subject to the most systemic strain, such as care homes and mental health hospitals. Neglect and individual wrongdoing involving vulnerable patients often stems from state-level failures in providing proper pay and conditions in these settings.^87^ Poor quality employment in certain health and care settings is very often a result of significant funding cuts where staff are doing more for less. Concerns have been raised about the criminalization of care workers when the cause often lies elsewhere. The same can be said of nurses working in these care contexts. Hayes argues, ‘poor quality care is a corollary of poor-quality employment’.^88^ Chronic underfunding of services can foster a form of *collective indifference* to harm^89^ or, as articulated by the Lampard Inquiry, *compassion fatigue*: a systemic erosion of empathy and care that occurs as professional resilience is progressively depleted.^90^ None of the neglect-type cases involving nurses in the dataset met the threshold for prosecution, yet cases of fatal harm and therefore MM investigations will likely rise due to further economic cuts. In fact, two of the three prosecutions for MM since 2019 involve nurses in such stretched state settings—a mental health ward manager and a police custody nurse—whereas only one of the prosecutions in the previous 11-year period involved a mental health nurse.^91^

In relation to the deceased in these cases, while there has been attention to disproportionality and inequality in relation to the race of doctors involved,^92^ there has been little attention to the characteristics of the deceased. Specific details on victim characteristics were not available in the data, but analysis of the type of case shows that some of the deaths highlight broader patterns of inequality in healthcare treatment that implicate race, age, disability, class and gender. These patterns extend beyond individual healthcare professionals or single healthcare trusts and their policies, reflecting much broader structural harm—namely, structural violence. Structural violence refers to indirect forms of harm built into the social, political, and economic structures of society that prevent individuals or groups from meeting their basic needs or reaching their full potential.^93^ At the heart of this violence lies structural inequality: intersections of class, ‘race’, gender, and broader systemic disparities generate conditions that, in their most extreme forms, result in premature and unnatural death,^94^ including deaths associated with fatal medical errors.

Three of the MM cases (1.56 per cent) involved healthcare professionals restraining Black men in mental health settings leading to death. Given the specificity of both the patient group and clinical context, this represents a disproportionate clustering of MM cases, suggesting an elevated risk of fatal outcomes and subsequent criminalization of HCPs in these settings. Indeed, these cases accord with recent data showing that the number of Black inpatients injured while being restrained in mental health units has risen dramatically—at the same time as the number of non-Black inpatients injured has fallen.^95^ These cases are also quite different from those involving complex clinical error that dominated the past MM debate. One case involved Olaseni Lewis, who sought help as a vulnerable voluntary patient and died after being restrained by up to 11 police officers, whilst healthcare staff failed to discharge their duty to him.^96^ There were also four cases of BAME women who died in gynaecology and obstetrics settings, reflecting wider trends of disproportionate deaths involving BAME women in these settings.^97^ Also, as will be shown below, over one third of all the cases involved vulnerable patients (including elderly patients, patients with dementia, mental health patients) in hospitals, care homes, secure hospitals, or other mental health settings where there was evidence of high levels of negligence and neglect, again reflecting the wider healthcare scandals discussed above that have identified serious failings in care for these specific groups.^98^ Another case in the dataset involved a death in custody (explored in the next section) where HCPs failed to act in accordance with their duty of care, reflecting research showing continued endemic inequalities in the provision of healthcare in prison and custody settings.^99^

Therefore, this analysis of the parties involved in MM cases uncovers previously overlooked disparities. In summary, nurses are almost as likely to be investigated and prosecuted as doctors, yet for different types of error, and they are less likely to challenge conviction, while many deaths in MM cases also reflect wider structural inequalities in healthcare. This evidences the need to go beyond current doctrinal analyses to more fully understand these cases through the lens of structural conditions.

### C. Type of error and culpability

The first part of this article showed that the majority of MM cases did not proceed to trial and that the types of error in this wider set of cases are relatively unknown in MM research. The second part explored who the victims and suspects are and demonstrated that some cases involve previously unexplored structural causes, rather than the more clinical or systemic factors emphasized in the literature. The aim of this section is to examine in greater depth the types of error present across all MM cases and, relatedly, where culpability for these errors arises, before turning in Section V to assess whether existing forms of accountability are adequate or whether reform is required.

As shown in Section II, in assessing culpability, it was not possible to access all evidence in all cases. Yet this section is not concerned with reassessing doctrinal correctness or the granular application of the GNM test. As shown earlier, the vast majority of cases were not prosecuted, and it is assumed, as other research into prosecutorial decision-making in this area has shown, that all tests were applied consistently on the evidence in the cases.^100^ Rather, I aim to discern the nature of error and where culpability broadly lies in order to consider where redress should focus.

The 192 cases were broken down into six main error types.

1. *Lack of timely diagnosis/treatment—*47 cases involved a failure to diagnose or treat in adequate time. Six prosecutions cases fell into this category.
2. *Neglect—*in 38 cases neglect was a factor in the death. Neglect is defined here as failure to follow basic care procedures. No prosecution.
3. *Error in administration of medication or blood*—27 cases. Three prosecuted cases fell into this category.
4. *Incorrect use of nasogastric or breathing tubes*—10 cases. One prosecuted case fell into this category.
5. *Surgical errors*—17 cases involved errors arising in surgery. Two prosecutions fell into this category.
6. *Errors in medication end of life care—*Five cases involved withdrawing or overdosing of medication in end-of-life care. These were categorized differently to prescribing errors as they involved end-of-life issues specifically. No prosecution.
7. *Other*—two additional categories did not fit the main 6—one in which the error was unknown from the details available (47) and one case involving organizational liability for *C. difficile* deaths that did not sit neatly in the above categories.

These error types were then sub-categorized into two broad types of crosscutting culpability within them. First, individual inadvertence (momentary errors without conscious awareness) and secondly, individual advertence (errors done with some conscious awareness, usually with some deliberate or unreasonable taking of risks and which most often are part of a course of action, thus distinct from momentary mistakes). These broadly align with the distinction Merry and Brookbanks make between ‘errors’/mistakes and ‘violations’/intentional deviations from standard safety practices.^101^ Violations include a situation where a HCP decided ‘to do something in the knowledge that the given action or decision will place at risk some aspect of safety or of the system’.^102^ There is a hazy line between the two. Advertence can be inferred under certain circumstances even if a HCP denies consciously taking a risk and claims inadvertence, for example if a HCP ignores or forgets (without reason) to adhere to well-known professional statutory guidance and/or it was part of an ongoing course of action where it was more likely conscious knowledge of risk would have arisen at some point.^103^ The analysis recognizes the extreme complexity in demarcating the line between the criminal thresholds of advertence and inadvertence.^104^ However, the aim here was not to assess if the cases did or should meet the standard for criminal negligence, where assessing the level of culpability required is indeed fraught with difficulty. Rather, I sought to identify where culpability broadly lay in order to allow a consideration of where redress should be broadly focused (eg, purely systemic or also individual) regardless of how far the case proceeded.

These two types of individual culpability (advertent and inadvertent) were crosscut with systemic fault (confined to individual institutional fault such as policies in place within a healthcare trust or care home) and structural fault (where the cause of the error goes beyond an institution to involve endemic issues involving multiple institutions often including state culpability as well as structural violence in relation to race, age, disability).

#### 1. Individual inadvertent error/systemic fault

In around half the cases analysed, an HCP made an inadvertent, momentary error—the type of error made without conscious control that generates anxiety amongst HCPs. Significantly, none of these types of inadvertent errors was prosecuted. These inadvertent errors were particularly prevalent in relation to failure to diagnose and treat (category 1), misplacement of nasogastric and breathing tubes (category 4), and errors in administration of drugs (category 3), where they made up around half of these categories. Such errors had clear links to systemic fault. Available evidence showed that prosecutorial consideration of these systemic issues was central in many of these types of cases, confirming past work on prosecutorial decision-making.^105^ One case involved fatal administration of penicillin to a patient who was allergic. There was clear evidence that the nurse involved had known but had forgotten about the allergy. While this was a clear negligent breach of duty, since evidence suggested a momentary error and it was recognized that the degree of understaffing and overcrowding of the ward at the time meant that the nurse was ‘intellectually and emotionally overloaded’, the case was not prosecuted, in line with CPS guidelines on applying MM offence criteria. Additionally, it is striking that many cases (12) involved the misplacement of nasogastric and breathing tubes. One such case involved ventilation equipment wrongly put together by nurses, leading to death. The equipment had been moved from its usual spot, which was central in making this a ‘one-off’ error. Again, the case did not proceed. Another patient suffocated when a ventilator was mishandled by inexperienced agency staff, and lack of training was identified as key to the error. Another case involved a 21-year-old hospice patient who suffocated when a nurse threw away part of the valve that should have been fitted to her breathing apparatus and subsequently ignored warnings from the machine. The inquest found staff were not trained in invasive ventilation and such a mistake would have been ‘quite easily done’.

Proposals in past MM research to shift blame away from individual inadvertence resulting from systemic fault are often focused on the few cases that have gone to court.^106^ The analysis of the above non-prosecuted inadvertent errors (half of the dataset) has value in being a discrete dataset demonstrating where there are patterns of systemic fault causing death and showing how learning needs to happen across this wider body of cases in the criminal justice system. This is taken up in Section V.

#### 2. Individual advertent errors/systemic and structural fault

The other half of the dataset of MM cases involved advertent errors. Within this, such advertent errors made up over half the cases in the second (neglect), fifth (error in administration of medication or blood), and sixth (surgical errors) types of medical error. In the neglect cases, well over half of these cases had high levels of individual culpability leading to the death of mental health patients, many detained under the Mental Health Act 1983, as well as vulnerable adults in care homes, and dementia patients within hospitals. As many recent reports into scandals in mental health hospitals and care settings have shown (and many of these MM cases were implicated in such scandals), such conduct is often deliberate,^107^ and this was visible in the data. Many MM cases involved HCPs ignoring or deliberately overriding safety systems in place in mental health settings; one case involved the failure to follow a number of safety protocols leading to a patient being allowed to access washing up liquid, which he drank; another patient died of malnutrition. The Lampard Inquiry has so far found serious concerns over individual misuse of restraint, and high levels of abuse, neglect and unsafe care in mental health hospitals.^108^ For example, undercover reporting exposed staff members sleeping on wards when they were supposed to be monitoring patients one-to-one.^109^ The care home and hospital setting neglect-type deaths involved similar types of advertent culpability, including a dementia patient who walked out of a dementia ward and was involved in a fatal road traffic incident. The nurse investigated was instrumental in allowing this—she had refused to do a staff handover on that day, meaning no one had known the patient was vulnerable and had gone missing—and subsequently lied about her failings.

Similarly, 8 out of the 17 cases in the fifth category, errors made in surgery, involved very high levels of individual advertence. For example, one case involved a surgeon who had double the normal death rate following errors in surgery. Concerns had been raised multiple times, prompting GMC restrictions on his practice. Such alerts surrounding previous suspicious deaths relating to one surgeon were common in this category. These cases can be said to involve ‘bad apple’ surgeons, who Mullock defines as ‘failing to deliver on their professional commitment’.^110^ They highlight the need for early intervention and how NHS whistleblowers are not listened to.^111^

Half of the cases in the sixth category involved wider healthcare scandals in end-of-life care, again entailing serious advertent conduct by individuals. One case involved hundreds of deaths and concerns about the use of diamorphine and syringe drivers that formed part of the inquiry at Gosport War Memorial Hospital centred on Dr Jane Barton.^112^ Like ‘bad apple’ surgeons, this case too showed a broader failure to identify patterns in deaths and how whistleblowers were ignored and unprotected when speaking out about more senior colleagues.^113^

Details of errors in the first (lack of timely diagnosis/treatment), third (errors in administration of medication or blood) and sixth categories (errors in medication in end-of-life care) demonstrate how they too depart from the one-off error made by an under-pressure doctor and involve high levels of advertent culpability. Half of the cases prosecuted (6 out of 12) fell into the first category, including some high-profile cases such as *Bawa-Garba*.^114^ Cases that did not reach trial in this first category revealed highly culpable errors, including, for example, the death in prison of 19-year-old Jordan Hullock, who died of meningitis. The inquest following the criminal investigation heard that Jordan was left to deteriorate over the course of days in appalling conditions in full view of staff, including healthcare staff.^115^ In the third category, there was a case of a nurse who gave the wrong fluid to a patient; this was not a momentary error but involved breaches of numerous strict Trust policies governing the administration of fluids. Moreover, this error was in a non-urgent situation where there were no other wider circumstances to indicate systemic fault or pressure. Finally, the deaths involving restraint of Black mental health patients involved individual HCPs who failed to intervene in situations where patients were in life-threatening situations. Olaseni Lewis was rendered unconscious after 45 min of restraint, yet this was not treated as a medical emergency by a nurse and doctor who both failed to intervene when police officers continued to restrain him.^116^

When analysing such advertent culpability, it is perhaps surprising that more cases did not proceed to prosecution. Furthermore, following *Rose* and *Sellu*,^117^ and the subsequent higher and clearer thresholds for prosecution, it seems unlikely that some cases prosecuted in the past would be now. This analysis shows how even where there are very high levels of individual culpability, MM is ill-suited to address the forms of error that characterize these cases. According to past research on prosecutorial decision-making, many of the above cases involved culpable conduct from a number of individuals which, when grouped together, may constitute ‘grossness’, but not when considered individually, and many cases fall on the causation limb.^118^ Despite not proceeding to prosecution, this body of previously unknown cases therefore reveals a great deal about fatal medical error and suggests that advertent errors constitute a higher proportion of MM cases than the past debate has assumed.

Such high levels of individual advertence, however, still have some links to systemic fault in the form of inadequate staff training, lack of coherent institutional policies, poor workplace cultures, and, of course, staff shortages. For example, in the case of Olaseni Lewis, the jury in the subsequent inquest highlighted failures in staff training around restraint. The Lampard Inquiry, which featured some MM cases, found numerous and interconnected systemic failures, particularly around staffing issues, and a culture of “institutional defensiveness” and failures to learn and act on recommendations that were raised by regulatory bodies like the Care Quality Commission (CQC).^119^ Similar systemic issues are likely to be prevalent in many of the other neglect-type cases discussed above in mental health settings and care homes. Nevertheless, the individual errors described above were often deliberate and wilful, so that they were not wholly mitigated by broader systemic issues.^120^

Finally, and as discussed in Section IV.B, many of the above cases of advertence not only contain elements of systemic fault but also feature wider structural violences, which is something that has not been addressed in previous MM research. The patterns of individual and systemic error often go beyond one particular healthcare trust or care setting to reveal more endemic and widespread harm linked to the state and structural violence. For example, the neglect-type cases above reveal issues around ‘collective indifference’ to harm in pressurized and underfunded sectors of mental health hospitals and care homes, going well beyond individual workers and institutions.^121^ Evidence being given to the Lampard Inquiry shows that the lack of therapeutic intervention in many cases is not an issue confined to Essex but a result of similar failings across the country, showing ‘the NHS at its worst’.^122^ There are also issues around classed and raced structural violence in prisons and hospitals that go beyond any particular healthcare professional, prison, or hospital. Many of the MM cases also show wider structural failures of regulators to prevent the fatal harms and that allow similar patterns of error on a national scale, for example, the failures of the Care Quality Commission (CQC) identified in the Lampard Inquiry.^123^

Regardless of where the cause ultimately lies, all these cases reveal cross-cutting serious levels of culpability which need to be addressed and require much wider forms of redress than those currently available or proposed.

## V. Beyond criminality: fatal medical errors, learning, and accountability

To recap, the MM cases confirmed many trends identified in previous research, while also challenging and dispelling some of the concerns that have arisen among HCPs and scholars. Prosecutions for the offence are rare, declining further in recent years. No cases in the sample involving inadvertent, momentary error combined with high levels of systemic culpability proceeded to prosecution. This confirms that, although subjective recklessness is not a requirement of the offence, cases are rarely prosecuted in its absence. Although elements of the offence remain highly contested and even low numbers of prosecutions understandably generate anxiety among HCPs,^124^ these concerns should be kept in proportion to the empirical reality.

The cases, however, also reveal newer, previously unexplored trends, particularly in relation to the cases that are not prosecuted. Cases involving momentary inadvertent fault revealed systemic errors that need to be learnt from, regardless of whether they result in prosecution; however, as discussed below, this does not always happen once an MM file is closed. Conversely, while some more advertent errors did result in prosecution, the vast majority did not, due to the high thresholds required for the offence. However, many still did involve high levels of individual culpability, and several also involved systemic fault, which often veered into structural violence. To deal with such cases, in previous co-authored work I have argued for the creation of a new offence of ‘medical neglect endangering life’, which would allow such fault to be prosecuted without the need to prove causation or meet the very high threshold of negligence required for gross negligence. However, following analysis of this broader range of cases, such a proposal is problematic. Individual errors were shown to be often intricately tied to systemic and structural violence. Therefore, introducing such an offence would perhaps address individual behaviour but would also contribute to the further criminalization of care workers and fail to tackle the broader systemic and structural causes of such fatal errors identified in the data. Seeking redress through criminalizing corporate negligence would also not be a complete solution: as stated, such prosecutions are extremely difficult and would only focus on systemic aspects, not individual or structural faults.^125^ The cross-cutting forms of culpability within the cases need an approach that goes beyond criminal law. As Tombs argues, ‘the lens of “crime” is a snapshot of an act or omission; it does not unpick the complex processes of power behind that’.^126^ Taking an approach outside of the criminal law would allow us to analyse the decisions made, policies implemented and cultures developed ‘so that we can then think of these in combination in terms of conditions, states of affairs, incubating phenomena and triggering events, and chains of processes’^127^ to allow more effective means of redress.

Of course, regardless of the outcome in the criminal justice system, MM cases will lead to several other investigations and routes to redress in wider regulatory fields, including patient safety mechanisms, clinical negligence, and professional regulation as well as other post-death investigations including inquests. It is likely many of these deaths will also be included in national patient safety data mechanisms.^128^ The worst and most prolific cases will have had public inquiries (including the Gosport and the Essex mental health cases). Scholarship on the regulation of patient safety is a well-established field and goes well beyond the scope of this article,^129^ yet three broad criticisms of all these regulatory (and semi-regulatory) processes can be discerned which mean that none of them can individually or collectively provide the accountability and learning that the fatal errors in MM cases reveal. First, the processes are fragmented. For example, professional regulation could result in sanctions against a HCP, yet its remit is not to address the wider systemic or structural causes of that HCP’s behaviour.^130^ Secondly, the processes also lack consistency; for example, numerous studies show the huge variability in the rate at which individual coroners produce PFDs,^131^ and a previous study has shown that many MM cases involving serious error are often signed off as ‘natural causes.’^132^ Additionally, under-reporting of PSIs is still an issue in patient safety data.^133^ Thirdly, in the processes where there is some form of mechanism for change, there is a lack of meaningful analysis and enforcement, so, for example, recommendations for change made in PFDs or national PSI alerts are most often not implemented.^134^ In the coronial system, there is a lack of a central, systematic approach to monitoring and evaluating findings and recommended actions from PFDs in cases of medical error, and there is a widespread lack of response to them.^135^ This lack of enforcement is endemic even following major reviews, including public inquiries, where it is widely recognized that the resulting recommendations from lengthy investigations frequently go unimplemented.^136^ These significant deficiencies in death oversight and in professional and patient safety regulatory frameworks are frequently overlooked in MM scholarship when non-criminal forms of redress, such as patient safety mechanisms, are put forward as substitutes for reliance on the offence.^137^

Considering the high levels of culpability identified in MM cases and the weaknesses in wider systems of death oversight and regulation, there is a case for treating cases of fatal medical error as a distinct category of data that can be used to address such gaps in learning and prevention in fatal cases. Death represents the most severe and objective form of outcome, demanding meaningful redress and resulting in more detailed legal investigations, including for MM and inquest. Although there is still inconsistency in such legal processes, they do more easily allow for aggregation of the very worst errors, and they contain wider details on the events than is available in many other kinds of learning mechanisms such as PSIs and professional hearings. Criminal investigations and PFDs offer unparalleled insights into deaths due to PSIs that are not offered by other safety data such as Serious Incident reports, Never Events and other data on the NRLS.^138^ Bremner and others argue that if PFD reports had been more efficiently collated and studied, many of the well-known healthcare and social care scandals could have been avoided, such as Gosport.^139^ If PFD reports, MM investigations, and other forms of data were triangulated, they would provide a more complete picture of the deaths that occur. For example, many MM cases involved deaths in mental health care, yet there is currently no national count of deaths of mental health patients aside from data on suicide, which excludes other causes of death among these patients, including the crosscutting sources of culpability identified above.^140^ Many of these deaths could have been prevented with better data aggregation and more systematic learning from inquest and MM investigations.^141^ Therefore, MM cases, used alongside other available sources, are a crucial source of data, yet need to be systematically aggregated and studied.

On a more concrete level, learning from such data and using it to prevent future death could be achieved by a national mechanism for learning. INQUEST reaffirms the argument made above that often little is changed by recommendations stemming from current post-death processes, which are often fragmented, piecemeal, and *ad hoc*. They are calling for a ‘National Oversight Mechanism’, which would be a public body responsible for collating data and following up on any recommendations arising from post-death processes in state-related deaths, including criminal investigations and also inquests, public inquiries and official reviews if they occur.^142^ Related to this, the State-related Deaths (National Oversight Mechanism) Bill is seeking to establish a body to monitor recommendations arising from investigations into state-related deaths.^143^ It would be a powerful tool for harm prevention if all MM investigations on fatal medical error, as well as any resulting inquests, potential inquiries and other data, were also collated into this centralized system of learning. Using such a mechanism to aggregate and triangulate the data from across the multiple investigations that typically follow a medical error death—and that often intersect with other types of state-related deaths—would expose the cross-cutting levels of individual, systemic and structural patterns of failure and result in more meaningful change and learning.

## VI. Conclusion

Narratives surrounding trends in the use of MM have led to much concern, prompting a powerful medical profession, along with many legal scholars, to oppose purported overcriminalization and advocate for reforms that shift liability onto systems rather than individuals. However, research has largely been focused on the doctrinal analysis of MM in a few appellate court cases involving clinical error. There has been a lack of attention to broader patterns in the cases that affect not only practitioners but also patients, who remain the overlooked victims of these errors. While the offence of GNM is contentious, and doctrinal debate will continue regarding the use of criminal negligence in healthcare cases, analysis needs to extend beyond application of the offence and its prosecution to focus on a broader spectrum of MM cases.

A fuller analysis of MM cases reveals substantial levels of harm and culpability, yet only a small proportion result in prosecution, highlighting a significant gap in accountability. MM cases show that many inadvertent and systemic clinical errors could be prevented through better data gathering and learning. Other MM cases involve advertent fault and structural abuse and neglect of mental health and care home residents, often widespread and increasingly common. Yet, these cases, which constitute a high proportion of all MM cases, have remained largely hidden and invisible in the doctrinal academic debates surrounding this contentious offence. Cowan and Wincott note, ‘socio-legal scholars have been encouraged to take back legal technicalities and use them for our own ends’. ^144^ In previous co-authored work, I demonstrated just how important attention to ‘the technical’ is for this debate, showing how it helps to unpick both the CPS’s use of criminal law doctrine and the ways in which the doctrine itself remains wanting.^145^ Yet, too much attention to the technical, and not enough empirical investigation of what MM cases involve, has led to what Riles describes as ‘legal knowledge cut off from the “social ends” it purports to instrumentalise’.^146^

To reconnect legal knowledge on MM to its social ends, analysis must extend beyond appellate decisions and move past both proposed and existing solutions, particularly those centred on criminal law. What is required instead are approaches capable of addressing, and preventing, the cross-cutting structural dimensions of culpability, alongside individual and systemic failures, in order to respond effectively to the complex and varied nature of fatal medical errors. One such mechanism would be a national accountability framework, though it would not be without limitations. Many of the MM cases show that prevention would only be possible through challenging deeper structural harms related to race, gender, age class, disability, as well as chronic underfunding and devaluing of certain sectors of healthcare provision such as mental health and social care. Whilst unpacking these issues is far more complex than this article can fully address, the first step is to broaden the analysis from doctrinal accounts of MM to situate all the cases in broader bodies of work on death oversight and regulation. Whatever the approach, the data presented here suggest a need to use the criminal law to go beyond the criminal law.

## Footnotes

1 Patient Safety Event Data Quarterly <https://www.england.nhs.uk/statistics/statistical-work-areas/patient-safety-data/2025-26/quarter-1-2025-26-april-may-june-2025/> accessed 1 June 2026.

2 Not all PSIs are human medical errors; some incidents happen without anyone making a mistake eg, failure of equipment.

3 Namely, if a death occurs that is unnatural, suspicious, or has occurred in the care of the state, then it should be referred to a Coroner. See E Kirkton-Darling, *Death, Family and the Law: The Contemporary Inquest in Context* (Bristol University Press 2022).

4 Adomako [1993] 4 All ER 935 CA; [1995] 1 AC 171, HL.

5 See O Quick, ‘Medicine, Mistakes and Manslaughter: A Criminal Combination?’ (2010) 69 Cambridge Law Journal 186; M Brazier and N Allen, ‘Criminalising Medical Malpractice’ in C Erin and S Ost (eds), *The Criminal Justice System and Healthcare* (OUP 2007) 15–28.

6 O Quick, ‘Prosecuting ‘Gross’ Medical Negligence: Manslaughter, Discretion, and the Crown Prosecution Service’ (2006) 33 Journal of Law and Society 421; O Quick, ‘Expert Evidence and Medical Manslaughter: Vagueness in Action’ (2011) 38 Journal of Law and Society 496; C Crosby, ‘Gross Negligence Manslaughter Revisited: Time for a Change of Direction?’ (2020) 84 Journal of Criminal Law 22.

7 D Griffiths and O Quick, ‘Managing Medical Manslaughter Cases: Improving Efficiency and Transparency?’ University of Bristol Law Research Paper Series, May 2019. See also Department of Health, *Gross Negligence Manslaughter in Healthcare: The Report of a Rapid Policy Review* (Department of Health 2018) para 4.4 (Williams Review).

8 For an overview, see J Samanta and A Samanta, ‘Gross Negligence Manslaughter and the Delivery of Healthcare: A Time for Change?’ (2019) 26 European Journal of Health Law 389. See also M Robson, J Maskill and W Brookbanks, ‘Doctors Are Aggrieved—Should They Be? Gross Negligence Manslaughter and the Culpable Doctor’ (2020) 84 Journal of Criminal Law 312.

9 M Brazier and others, ‘Improving Healthcare Through the Use of “Medical Manslaughter”? Facts, Fears and the Future’ (2017) 22 Journal of Patient Safety Risk Management 88.

10 Eg, see A Alghrani and others, ‘Balancing the Scales of Safety: The Criminal Law’s Impact on Patient Safety and Error Reduction’ (2024) 62 British Journal of Oral and Maxillofacial Surgery 229; E Olvaardt, ‘Blame Does Not Keep Patients Safe’ (2019) 32 Community Eye Health 36; A Samanta and J Samanta ‘Death Caused by Negligent Medical Care: Reconsidering the Role of Gross Negligence Manslaughter in the Aftermath of Bawa-Garba’ (2021) 21 Medical Law International 293.

11 For recent accounts, see J Lilleker, ‘A Betrayal of Trust? Back to the Drawing Board for Medical Manslaughter’ (2021) 85 Journal of Criminal Law 335; B Bartlett, ‘The Possibility of AI-induced Medical Manslaughter: Unexplainable Decisions, Epistemic Vices, and a New Dimension of Moral Luck’ (2023) 23 Medical Law International 241.

12 Most recently these cases have been *Sellu v R* [2016] EWCA Crim 1716l; *Bawa-Garba v R* [2016] EWCA Crim 1841; *Rose v R* [2017] EWCA Crim 1168.

13 There are some exceptions; eg, see AM Farrell, A Alghrani and M Kazarian, ‘Gross Negligence Manslaughter in Healthcare: Time for a Restorative Justice Approach?’ (2020) 28 Medical Law Review 526. However, even within this, concern is centrally about fairness for HCPs.

14 There have been some empirical studies examining MM, particularly in relation to prosecutorial decision making, beginning Quick’s (n 6) interviews with crown prosecutors as well as CPS case file analysis see D Griffiths and A Sanders, ‘The Road to the Dock: Prosecution Decision-Making in Medical Manslaughter Cases’ in D Griffiths and A Sanders (eds), *Bioethics Medicine and the Criminal Law* (CUP 2013) 117–158. However, due to restrictions on access to data, this work has relied on small, unrepresentative samples.

15 This draws on but significantly extends work done by Griffiths and Quick (n 7).

16 Griffiths and Quick (n 7).

17 ibid.

18 ibid.

19 ibid.

20 ibid.

21 I Convery and D Cox, ‘A Review of Research Ethics in Internet-based Research’ (2012) 6 Practitioner Research in Higher Education 50.

22 Griffiths and Sanders (n 14).

23 ibid.

24
*Adomako* (n 4).

25 ibid 187.

26 For example, see J Horder, ‘Gross Negligence and Criminal Culpability’ (1997) 47 University of Toronto Law Journal 495. See also (n 5).

27 Although subjective recklessness is not required, it is not irrelevant, see *R (Rowley) v DPP* [2003] EWHC 693. See Griffiths and Sanders (n 14) for a detailed discussion of how cases are rarely charged without some evidence of subjective recklessness.

28 Quick (n 6).

29 J Vaughan, O Quick and D Griffiths, ‘Medical Manslaughter: Where Next?’ (2018) 100 Bulletin of the Royal College of Surgeons of England 251.

30
*Rose* (n 12).

31 ibid [77] per Leveson P.

32 For a discission of this see A Mullock, ‘Gross Negligence (Medical) Manslaughter and the Puzzling Implications of Negligent Ignorance: Rose v R [2017] EWCA Crim 1168’ (2018) 26 Medical Law Review 346, 353.

33 EWCA Crim 1093 [5].

34 F Stark, ‘In Praise of Rose’ (2019) 8 Archbold Review 7.

35 ibid 10.

36
*Sellu* (n 12).

37 Crown Prosecution Service, Gross Negligence Manslaughter Legal Guidance <https://www.cps.gov.uk/legal-guidance/gross-negligence-manslaughter> accessed 20 April 2026.

38 ibid.

39 Stark (n 34).

40 ibid 8.

41 K Laird, ‘Case Comment: Manslaughter: R v Rose’ (2018) 1 Criminal Law Review 76.

42 Mullock (n 32) 353.

43 Laird (n 41).

44 R Ferner and SE McDowell ‘Doctors Charged with Manslaughter in the Course of Medical Practice 1795-2005’ (2006) 99 Journal of the Royal Society of Medicine 309.

45 Williams Review (n 7); Griffiths and Quick (n 7).

46 For one of the earliest and leading versions of this argument see O Quick, ‘Medicine, Mistakes and Manslaughter: A Criminal Combination?’ (2010) 69 Cambridge Law Journal 186; See also A Lodge, ‘Gross Negligence Manslaughter on the Cusp: The Unprincipled Privileging of Harm Over Culpability’ (2017) 81 Journal of Criminal Law 125 and Mullock (n 32) 356.

47 Griffiths and Sanders (n 14) see also O Quick (n 6).

48 Mullock (n 32) 352 and Crosby (n 6).

49 Lodge (n 46) 138.

50
*Bawa-Garba* (n 12).

51 For an overview, see Samanta and Samanta (n 5). See also S Ost, ‘Drs Bramhall and Bawa-Garba and the Rightful Domain of the Criminal Law’ (2019) 45 Journal of Medical Ethics 151.

52
*Bawa-Garba v General Medical Council & Ors* [2018] EWCA Civ 1879.

53 ibid [74].

54 Samanta and Samanta (n 10).

55 Ibid. See also J Lilleker, ‘A Betrayal of Trust? Back to the Drawing Board for Medical Manslaughter’ (2021) 85 Journal of Criminal Law 335.

56 Griffiths and Sanders (n 14). See also O Quick, ‘Medical Manslaughter–Time for a Rethink?’ (2017) 85 Medico-Legal Journal 173, 180.

57 Bawa Garba (n 52 at para 75).

58 Alghrani and others (n10).

59 Brazier and others (n 9).

60 The Williams Review (n 7).

61 General Medical Council, Independent Review of Gross Negligence Manslaughter and Culpable Homicide, June 2019, 5 <www.gmc-uk.org/-/media/documents/independent-review-of-gross-negligence-manslaughter-and-culpable-homicide—final-report_pd-78716610.pdf> accessed 31 March 2026 (Hamilton Review).

62 Laird (n 39).

63 R Wheeler and R Wheeler, ‘Betrayal of Trust in Medical Manslaughter’ (2019) 83 Journal of Criminal Law 489, 501.

64 Lilleker (n 53) 8.

65 Laird (n 41).

66 Mullock (n 32).

67 The Hamilton Review (n 61) 71.

68 ibid 71.

69 For example, many academic articles reference just doctors within the title, for example, Robson and others (n 8).

70 Griffiths and Sanders (n 14).

71 Griffiths and Quick (n 7).

72 Dr Isyaka Mamman pleaded guilty to GNM in 2022. He attempted a rare and highly dangerous way of extracting a bone marrow sample despite desperate pleas from the victim and her husband to stop. The second case involved Benjamin Aninakwa who was found not guilty of GNM in 2025 but guilty of a Health and Safety Offence. This followed the death of Alice Figueiredo, a 22-year-old mental health patient. This case also involved a corporate manslaughter charge against Northeast London NHS Foundation Trust which was subsequently found not guilty. There is a third ongoing prosecution involving Sean Cregg who is charged with MM following the death of a man in police custody.

73
*Rose* and *Sellu* (n 12).

74 Griffiths and Quick (n 7).

75 ibid.

76 Samanta and Samanta (n 9). Though note the recent prosecution of a Hospital Trust see (n 72).

77 Nine of these cases are detailed in Vaughan, Quick and Griffiths (n 29). The CPS data contained three other earlier cases of Priya Ramnath, Sudhanshu Garg and Bala Kovvali.

78 Quick (n 5).

79 A Alghrani and H Saad, ‘Medical Manslaughter’ in Elizabeth Chloe Romanis, Sabrina Germain, and Jonathan Herring (eds), *Diverse Voices in Health Law and Ethics* (Bristol University Press 2025).

80 Hamilton Review (n 61) 27.

81 Griffiths and Sanders (n14).

82 Department of Health, ‘NHS Workforce’ <https://www.ethnicity-facts-figures.service.gov.uk/workforce-and-business/workforce-diversity/nhs-workforce/latest/> accessed 2 November 2025.

83 GMC Watch, ‘The Shocking Story of David Sellu’ <https://gmc-watch.com/dr-david-sellu/f/the-story-of-david-sellu> accessed 2 November 2025.

84 C Dyer, ‘Bawa-Garba Is Free to Practise Again without Restrictions after Tribunal Ruling’ (2021) 374 British Medical Journal 374.

85 N Hodson, ‘Regulatory Justice following Gross Negligence Manslaughter Verdicts: Nurses/Doctors Differences’ (2020) 27 Nursing Ethics 247.

86 ibid 250.

87 D Norris, D Baker and GC Richards, ‘Investigating Deaths of People with Autistic Spectrum Disorder in Health and Social Care in England and Wales’ (2025) 27 Journal of Adult Protection 40.

88 L Hayes, ‘Criminalising Care Workers: A Critique of Prosecution for Ill-treatment or Wilful Neglect’ in A Bogg (ed), *Criminality at Work* (Oxford Academic 2020) 290. See also J Herring *Vulnerable Adults and the Law* (OUP 2016).

89 Norris and others (n 87) 48.

90 The Lampard Inquiry is a statutory investigation into thousands of deaths of mental health inpatients under the care of NHS Trust(s) in Essex between 1 January 2000 and 31 December 2023. See <https://lampardinquiry.org.uk/> Some of the MM cases involved some of these deaths.

91 See (n 72).

92 Alghrani and Saad (n 79).

93 J Galtung, ‘Violence, Peace, and Peace Research’ (1969) 6 Journal of Peace Research 167.

94 S Tombs, ‘Justice After Grenfell? Crime, Harm and Structural Inequality’ (2025) British Journal of Criminology 1.

95 INQUEST, ‘Jury Condemns Police Restraint of Young Black Man in Mental Health Hospital Whilst Medical Staff Looked On’ <https://www.theguardian.com/uk-news/2024/feb/17/black-mental-health-patients-more-likely-injured-police-england> accessed 2 November 2025.

96 See Inquest, ‘Olaseni Lewis Inquest Conclusion’ <https://www.inquest.org.uk/seni-lewis-conclusion>

97 The Kings Fund, ‘The Health of Women from Ethnic Minority Groups in England’ <https://www.kingsfund.org.uk/insight-and-analysis/long-reads/the-health-of-women-from-ethnic-minority-groups-england> accessed 2 November 2025.

98 Norris and others (n 87).

99 K McLintock and L Sheard, ‘Prison Healthcare in England and Wales is in Perpetual Crisis’ (2024) 384 British Medical Journal 562. See also (n 72) for details of the current prosecution against a police custody nurse.

100 Griffiths and Sanders (n 14) was the only other analysis of MM CPS case files—based on a much smaller and unrepresentative sample. This research showed consistency in how the tests were applied on the evidence in cases. While it is recognized this might not be the case for this larger sample of cases, the main aim is to assess broad levels of culpability and types of error in order to assess how they should be dealt with regardless of whether there was a decision to prosecute. Therefore, any potential inconsistency in prosecutorial decision making is irrelevant for the main argument of the article.

101 See A Merry and W Brookbanks, *Merry and McCall Smith’s Errors, Medicine and the Law* (2nd edn, CUP 2017).

102 Robson and others (n 8) 326. See also Lodge (n 46).

103 ibid.

104 ibid. See also A Ashworth and J Horder, *Principles of Criminal Law* (7th edn, OUP 2013) 181–185.

105 Griffiths and Sanders (n 14).

106 For example, Alghrani and others (n 10) and Alghrani and Saad (n 79).

107 Lampard Inquiry (n 90).

108 C Dyer ‘Essex Mental Health Inquiry Will Uncover More Deaths, Says Chair’ (2024) 386 British Medical Journal q1991.

109 Care Quality Commission, ‘Essex Partnership University NHS Foundation Trust Improvements Are Needed on Acute Wards’ <https://www.cqc.org.uk/press-release/cqc-tells-essex-partnership-university-nhs-foundation-trust-improvements-are-needed> accessed 3 November 2025.

110 A Mullock ‘Surgical Harm, Consent, and English Criminal Law: When Should ‘Bad-Apple’ Surgeons Be Prosecuted?’ (2021) 21 Medical Law International 343.

111 JV McHale ‘Whistleblowing in the NHS: The Need for a New Generation to Learn the Lessons’ (2022) 48 Journal of Medical Ethics 684.

112 Gosport Independent Panel (2018) <https://www.gosportpanel.independent.gov.uk/> accessed 1 November 2025.

113 McHale (n 111).

114
*Bawa Garba* (n 12).

115 INQUEST, ‘Teenager Whose Meningitis and Heart Condition Was Overlooked Was Failed by HMP Doncaster Healthcare Staff, Inquest Finds’ <https://www.inquest.org.uk/jordan-hullock-closes> accessed 1 November 2025.

116 See (n 96).

117 See (n 12).

118 Griffiths and Sanders (n 14).

119 Lampard Inquiry (n 90). See also (n 87).

120 See (n 56) for a discussion of this.

121 Norris and others (n 87).

122 Lampard Inquiry (n 90).

123 ibid. The Lampard Inquiry has exposed significant, long-standing criticisms of the CQC and broader healthcare regulatory systems.

124 Debate will continue around how to resolve the ongoing uncertainty surrounding the tests in GNM in a way that ensures fairness for HCPs and others subject to them. While proposals to change the test in GNM to one of subjective recklessness have merit in that it would clarify the required standard, other proposals that give greater weight to victims’ interests are also compelling; see R Wheeler and R Wheeler, ‘Betrayal of Trust in Medical Manslaughter’ (2019) 83 Journal of Criminal Law 489.

125 J Hébert, S Bittle and S Tombs ‘Obscuring Corporate Violence: Corporate Manslaughter in Action’ (2019) 58 Howard Journal of Crime and Justice 554. The use of financial penalties against NHS organizations for corporate and health and safety breaches also presents inherent tensions, see Samanta and Samanta (n 10 at 306).

126 S Tombs, ‘Grenfell: The Unfolding Dimensions of Social Harm’ (2019) 3 Justice, Power and Resistance 61, 61.

127 ibid 62.

128 The need to tackle avoidable harm and make significant improvements to the safety of health and social care is very well-established, see Learn from Patient Safety Events service <https://www.england.nhs.uk/patient-safety/patient-safety-insight/learning-from-patient-safety-events/learn-from-patient-safety-events-service/> accessed 4 November 2025.

129 For a detailed examination see O Quick, *Regulating Patient Safety: The End of Professional Dominance?* (CUP 2017).

130 Through the Medical Act 1983.

131 BT Bremner and others, ‘A Systematic Narrative Review of Coroners’ Prevention of Future Deaths Reports (PFDs): A Tool for Patient Safety in Hospitals’ (2023) 28 Journal of Patient Safety and Risk Management 227, 234.

132 Griffiths and Sanders (n 14).

133 For example, see A Meslamani, ‘Underreporting of Adverse Drug Events: A Look into the Extent, Causes, and Potential Solutions’ (2023) 22 Expert Opinion on Drug Safety 351.

134 S Jeraj, ‘Preventing Future Deaths: What Have We Learnt from Reports into Preventable Fatalities?’ (2024) 386 British Medical Journal 1943.

135 See the Preventable Deaths Tracker which highlights the rate at which organizations fail to respond to PFD reports <https://preventabledeathstracker.net/about/> accessed 20 November 2025. See also Patient Safety Learning, ‘The Patient-Safe Future: A Blueprint For Action’ <https://s3-eu-west1.amazonaws.com/ddme-psl/content/A-Blueprint-for-Action-240619.pdf?mtime=20190701143409> accessed 20 November 2025.

136 D Elliott and M McGuinness ‘Public Inquiry: Panacea or Placebo?’ (2002) 10 Journal of Contingencies and Crisis Management 14. For a review of the literature on public inquiries and specifically some of the indirect ways inquiries can promote change, see D Goodwin and others, ‘What We Do and Do Not Know about Public Inquiries: A Narrative Review’ (2025) 20 Contemporary Social Science 713. See also I Loader, ‘A Question of Sacrifice: The Deep Structure of Deaths in Police Custody’ (2020) 29 Social and Legal Studies 40.

137 Alghrani and others (n 10).

138 Bremner (n 131).

139 ibid 236.

140 D Baker, M Fidalgo and L Harrison-Brant, ‘Counting and Accounting for Mental Health Related Deaths in England and Wales’ (2024) 13 International Journal for Crime, Justice and Social Democracy 58, 58.

141 Many of the Essex Trusts mental health cases were investigated by the police for MM but closed due to not reaching the threshold. For example, see <https://www.eadt.co.uk/news/21398357.family-intelligent-son-died-nhs-care-join-mums-fight-inquiry/> accessed 1 November 2025.

142 INQUEST, ‘No More Deaths’ <https://www.inquest.org.uk/Handlers/Download.ashx?IDMF=b480f898-7fbd-4c9c-a948-50dd3fad3a04> accessed 1 May 2026.

143 This would be a body to monitor recommendations arising from investigations into contentious deaths and would have powers to require action in relation to such recommendations. See <https://bills.parliament.uk/bills/3837> accessed 1 July 2026.

144 D Cowan and D Wincott (eds), ‘Exploring the “Legal”’ in *Exploring the ‘Legal’ in Socio-Legal Studies* (Palgrave Macmillan) 1.

145 A Sanders and D Griffiths, ‘Following the Law or Using the Law? Decision-Making in Medical Manslaughter’ in D Cowan and D Wincott (eds), *Exploring the ‘Legal’ in Socio-Legal Studies* (Palgrave Macmillan 2016) 225–244.

146 A Riles, ‘A New Agenda for the Cultural Study of Law: Taking on the Technicalities’ (2005) 53 Buffalo Law Review 973.

